# Gastric Signet-Ring Cell Carcinoma That Presented as an Elevated Lesion due to Fibromuscular Obliteration in the Lamina Propria

**DOI:** 10.1155/2021/2887256

**Published:** 2021-11-24

**Authors:** Yoshitsugu Misumi, Shin Ichihara, Kouichi Nonaka, Hiromi Onizuka, Yoji Nagashima

**Affiliations:** ^1^Department of Digestive Endoscopy, Tokyo Women's Medical University Hospital, 8-1, Kawada-cho, Shinjuku-ku, Tokyo 162-8666, Japan; ^2^Department of Surgical Pathology, Sapporo-Kosei General Hospital, 8-5, Kita 3 Jouhigashi, Chu-o-ku, Sapporo, Hokkaido 060-0033, Japan; ^3^Department of Surgical Pathology, Tokyo Women's Medical University Hospital, 8-1, Kawada-cho, Shinjuku-ku, Tokyo 162-8666, Japan

## Abstract

The widespread use of *Helicobacter pylori* eradication therapy in recent years has reduced the *H. pylori* infection rate, indicating that gastric cancer cases diagnosed in the future may be *H. pylori*-naïve. The typical endoscopic presentation of signet-ring cell carcinoma, which accounts for the majority of *H. pylori-*naïve gastric cancer cases, is a discolored, flat, or depressed lesion; it is rarely presented as an elevated lesion. In this study, we treated a patient with elevated signet-ring cell carcinoma in an *H. pylori-*naïve stomach. Histopathological testing after endoscopic submucosal dissection showed proliferation of fibromuscular tissue in the tumor, which may have caused the formation of the elevated lesion.

## 1. Introduction

In the past, most cases of gastric cancer occurred in the stomach of people infected with *Helicobacter pylori* [1]. However, the widespread use of *H. pylori* eradication therapy in recent years has reduced the *H. pylori* infection rate, indicating that higher rates of *H. pylori*-naïve gastric cancer cases may be diagnosed in the future. *H. pylori-*naïve gastric cancer is broadly divided into three types: signet-ring cell carcinoma (SRCC), fundic gland-type gastric carcinoma, and foveolar-type gastric carcinoma [2]. Amongst these, SRCC reportedly accounts for over 90% of the cases [3].

The World Health Organization Classification of Tumors classifies all carcinomas consisting of discohesive cells arranged singly or in small clusters and lacking well-formed glands as poorly cohesive carcinoma. Cancer that mainly exhibits a signet-ring morphology is designated as SRCC [4]. Gastric SRCC is typically observed via endoscopy as a discolored lesion in the middle and lower gastric corpus of relatively young persons [3]. Upon gross inspection, most lesions can be seen as flat or flat-depressed, and elevated lesions are rare [5, 6]. However, a few reports of elevated undifferentiated gastric carcinoma mention its histopathological characteristics [5]. Here, we report a case of a small flat-elevated SRCC, which was curatively resected by endoscopic submucosal dissection (ESD), and discuss the pathology of its flat-elevated appearance.

## 2. Case Report

A 63-year-old female with no relevant medical history underwent upper gastrointestinal endoscopy for screening. She neither smoked nor drank alcohol and had no family history of malignancy. The patient's *H. pylori* serology result (IgG < 3) and fecal *H. pylori* antigen were negative. The patient had not undergone *H. pylori* eradication therapy.

**Figure 1:**
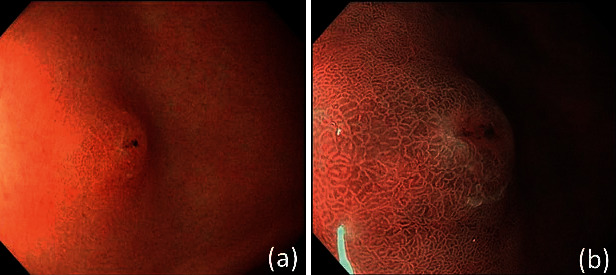
(a) A 5 mm poorly demarcated pale-red elevated lesion observed in the anterior wall of the antrum against the background of nonatrophic *H. pylori*-negative mucosa. (b) Swelling of the glandular structures is present, although the demarcation line is unclear.

Endoscopy revealed a 5 mm poorly demarcated pale-red elevated lesion with a slight depression at the top against the background of a nonatrophic *H. pylori*-negative mucosa (Figure 1). The demarcation line of the elevated lesion was also unclear under narrow-band imaging, but the swollen glandular structure of the elevated portion was clearly observed (Figure 1). The biopsy results of the elevated lesion confirmed that it was SRCC. As the lesion was small and neither thickened nor hard, it was diagnosed as intramucosal carcinoma. We finally assumed the lesion to be intramucosal gastric cancer composed of SRCC with 0-IIa + IIc according to the Paris classification; thus, endoscopic submucosal dissection was performed.

**Figure 2:**
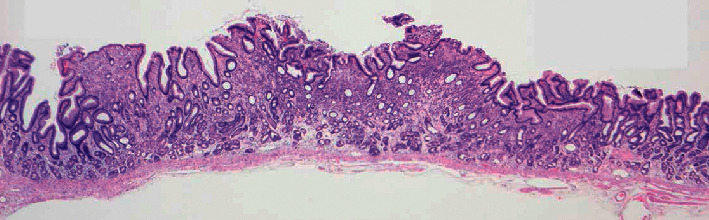
Cancer cells seen mainly invading the height of the glandular neck zone, with none in the deep mucosa. There is neither erosion of the surface epithelium nor any signs of tumor cell exposure on the surface. This is a flat-elevated lesion proliferating within the mucosa.

The resected specimen measured 28 × 18 mm and the lesion 8 × 5 mm. Histopathological investigations revealed signet-ring cell invasion at the tip of the elevated lesion and in the surrounding lamina propria. Layered cancer cells were mainly present at the height of the glandular neck zone; none were detected in the deep portion of the mucosa. In the areas where cancer cells were present, the foveolar epithelium of the surface layer showed mild hyperplasia and dilation of the intervening part between the crypts. However, there was no erosion of the surface epithelium, and no signs of direct tumor cell exposure on the surface. Hence, it was confirmed as a flat-elevated lesion proliferating within the mucosa with negative lymphovascular invasion and clear resection margins (Figure 2).

**Figure 3:**
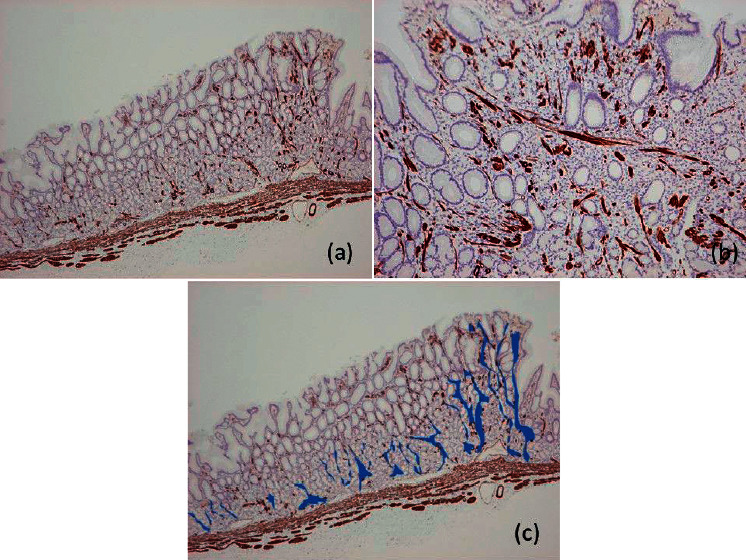
(a) Fibromuscular obliteration by *α*-smooth muscle myosin observed around the pyloric glands in the background mucosa surrounding the lesion; in places, this extends as if to support the foveolar epithelium. (b) Inside the lesion, fibromuscular obliteration seems to support the foveolar epithelium. (c) Schema representing fibromuscular obliteration. Fibromuscular obliteration is surrounded by blue paint.

In the background mucosa surrounding the lesion, immunohistochemistry by anti-*α*-smooth muscle actin revealed thin muscular bundles in the lamina propria. Fibromuscular obliteration extended from the muscularis propria to the lamina propria, as if to support the mucosal structure, and thickened the mucosa (Figures 3 and 3). Furthermore, a similar phenomenon was demonstrated inside the cancerous lesion and seemed to support the superficial foveolar epithelium (Figure 3). The resection was concluded as curative, and upper gastrointestinal endoscopy follow-up was scheduled 12 months after the ESD.

## 3. Discussion

Gastric SRCC is usually flat or depressed; however, there are rare cases of elevated lesions that have been successfully resected [5]. Two causes of elevated lesions include upwards tumor growth into the gastric lumen and coexistent desmoplastic or fibrotic reaction [7]. However, neither of these reasons was applicable in the case we report here. Instead, a fibromuscular obliteration was observed both inside and outside the lesion. Fibromuscular obliteration is seen in conditions such as mucosal prolapse syndrome (MPS) of the rectum and occurs when smooth muscle bundles extend from the muscularis mucosa into the lamina propria [8]. As the patient exhibited strong peristalsis, which was so frequent that it was difficult to take clear pictures, it is possible that a previously slightly depressed lesion was pushed out by intense peristaltic movements that led to fibromuscular obliteration of the lamina propria, consequently lifting the mucosa within the lesion to give it an elevated appearance. Additionally, the distribution of the SRCC in the present case was mostly limited to the glandular neck zone, and the absence of surface erosion indicated that no remarkable depression or step was formed, which may have also contributed to the observation of the lesion mainly as an elevation.

Although this report is just one case and further research is essential, we suggest the term “fibromuscular elevation” (FE) to describe the phenomenon by which fibromuscular obliteration, induced by peristalsis, elevates both tumor and nontumor tissue towards the luminal side. Similar to the present case, there is a report that the same “FE” phenomenon was observed in some differentiated adenocarcinoma [9]. Although this is a rare case, the possibility of undifferentiated carcinoma exhibiting FE should be considered when an elevated lesion is encountered in the antrum of an *H. pylori-*naïve stomach. In addition, it is difficult to differentiate the lesion from the raised erosions commonly seen in the antrum based on only endoscopic findings. Hence, a biopsy is mandatory when a single lesion is seen in the antrum.

## Data Availability

The data that support the findings of this study are available from the corresponding author upon request.
